# Analysis of genotype–phenotype correlation in patients with α‐thalassemia from Fujian province, Southeastern China

**DOI:** 10.1002/jcla.24696

**Published:** 2022-09-13

**Authors:** Yali Pan, Meihuan Chen, YanHong Zhang, Min Zhang, Lingji Chen, Na Lin, Liangpu Xu, Hailong Huang

**Affiliations:** ^1^ Medical Genetic Diagnosis and Therapy Center of Fujian Maternity and Child Health Hospital College of Clinical Medicine for Obstetrics & Gynecology and Pediatrics Fujian Medical University, Fujian Provincial Key Laboratory of Prenatal Diagnosis and Birth Defect Fuzhou China; ^2^ Medical Technology and Engineering College of Fujian Medical University Fuzhou China; ^3^ Fujian University of Traditional Chinese Medicine Fuzhou China

**Keywords:** gene diagnosis, genotype, novel gene mutation, phenotype, α‐thalassemia

## Abstract

**Background:**

There is a high carrying rate of α‐thalassemia in Fujian province. However, there are few large‐scale studies on the correlation between genotype and phenotype in Fujian province. The purpose of this study was to analyze the phenotype and genotype in a cohort of 2923 patients with α‐thalassemia in Fujian province, so as to provide reference data for screening and diagnosis of α‐thalassemia in Fujian province.

**Methods:**

The genotype of α‐thalassemia was detected by PCR reverse dot blot assay, gap‐PCR, single PCR, nested PCR, and sequencing. Clinical and hematological indices of 2923 patients were collected, and the correlation between genotype and phenotype was analyzed.

**Results:**

Among 10,350 patients, 2923 cases were found with α‐thalassemia, with a detection rate of 28.24%. Among them, ‐‐^SEA^/αα was the most common genotype, accounting for 64.80%. In addition, rare α‐thalassemia genotypes were detected in Fujian province, including ‐‐^THAI^/αα (0.41%), HKαα/‐‐^SEA^ (0.03%), and the novel α‐thalassemia gene mutation CD5 (GCC>ACC) (HGVS named HBA1: c.16G>A) (0.03%). Patients with deletional genotypes of α‐thalassemia were found to have higher RBC and lower Hb, MCV, MCH, and HbA2 than patients with non‐deletional genotypes of α‐thalassemia (*p* < 0.05).

**Conclusion:**

The clinical phenotype of α‐thalassemia is influenced by molecular mechanisms. HBA1: c.16G>A mutation is a novel mutation that was first reported in Fujian province, which enriches the human hemoglobin mutation spectrum.

## Graphical abstract

There were double peaks at nt224, G>A, in HBA1, corresponding to CD5 (GCC>ACC). It was named HBA1: c.16G>A using the Human Genome Variation Society (HGVS) nomenclature. HBA1: c.16G>A is a novel mutation that was first reported in Fujian province. The discovery of this novel mutation in α‐globin gene has enriched the database of hemoglobin variants, and the detailed genetic analysis and clinical symptom description of this mutation will contribute to the further study of hemoglobin function in the future.

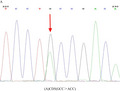

## 1 INTRODUCTION

α‐thalassemia is one of the most common autosomal recessive genetic diseases in the world. Its pathogenesis is due to the defect of α‐globin gene; the synthesis of α‐globin peptide chain was partially or completely inhibited, resulting in hereditary hemolytic anemia. It is one of the most common monogenic diseases with the highest incidence in the world and has attracted extensive attention at home and abroad because of its fatal and disabling nature, which can lead to birth death or birth defects.^1^ Around 80%–90% of α‐thalassemia is caused by genomic deletion of the α‐globin gene cluster on chromosome 16p13.3.^2^ There is increasing evidence that around 7.0% of the world's population are carriers of mutations in the α‐globin gene.^3^ α‐thalassemia has obvious regional or population differences and is mainly found in the Mediterranean, Africa, and Southeast Asia. The incidence of α‐thalassemia is high in southern China, especially in Sichuan, Yunnan, Guangxi, Guizhou, Guangdong, and Fujian.^4^ Fujian province, located in the southeast coastal area of China, is a province with high incidence of α‐thalassemia.^5^ At present, there is no other specific treatment for severe α‐thalassemia except long‐term blood transfusion or hematopoietic stem cell transplantation. Therefore, understanding the prevalence of α‐thalassemia in high incidence areas, monitoring the population of childbearing age, preventing the birth of children with severe α‐thalassemia, and gradually eliminating the prevalence of α‐thalassemia in the population through genetic intervention are the only coping methods.

However, there are few large‐scale studies on the correlation between genotype and phenotype in α‐thalassemia patients in Chinese population. In this study, 10,350 patients from Fujian province were analyzed for genotype and phenotype. Such a study may provide more data for genetic counseling and clinical diagnosis in this region.

## 2 METHODS

### 2.1 Ethics statement

This study was reviewed and approved by the Ethics Review Committee of Fujian Maternity and Child Health Hospital College of Clinical Medicine for Obstetrics & Gynecology and Pediatrics, Fujian Medical University. Signed informed consent was obtained from all participants following a detailed description of the purpose of the study. All experiments were performed in accordance with relevant guidelines and regulations.

### 2.2 Subjects and hematological indicator detection

From January 2019 to November 2021, peripheral blood samples of patients who underwent thalassemia‐related examinations in outpatients and inpatients of Fujian Maternity and Child Health Hospital College of Clinical Medicine for Obstetrics & Gynecology and Pediatrics, Fujian Medical University were collected. A total of 10,350 patients (3407 males and 6943 females) with household registration in Fujian province were screened, including 2923 cases of α‐thalassemia. α‐thalassemia was confirmed by molecular analyses. Information about patients diagnosed with α‐thalassemia was collected, and their genotypes and phenotypes were statistically analyzed. The median age was 27 (8.31) years. The median age was 5.5 (1.30) years for males and 28 (25.31) years for females. Exclusion criteria are as follows: (1) β‐thalassemia; (2) other blood diseases such as iron deficiency anemia and megaloblastic anemia; (3) malignant tumor; (4) systemic immune disorders; and (5) allergic constitution. Peripheral blood samples were collected from selected subjects and anticoagulated with EDTA‐K2. Approximately 2 ml of the anticoagulated blood sample was used for analysis of blood cell parameters on a Sysmex XN‐2000 automatic hematology analyzer (Sysmex; Shanghai, China), and the hemoglobin components and levels were analyzed using an automated capillary electrophoresis system (CapillaryS 2, software version 6.2; Sebia, Paris, France). Serum ferritin (SF) concentration, as a measure of iron status, was determined by the chemiluminescent microparticle immunoassay (CMIA) (Abbott; ARCHITECT ci16200, USA).

### 2.3 Common genotype test

DNA was extracted using the DNA Blood Extraction Kit (Yaneng Biosciences, Shenzhen, China). The deletions (‐‐^SEA^/, ‐α^3.7^/, and ‐α^4.2^/) and the mutations (α^CS^α/, α^QS^α/, and α^WS^α/) of α‐thalassemia were analyzed by PCR reverse dot blot assay using commercial kits (Yaneng Biosciences, Shenzhen, China) as described. The 17 common β‐thalassemia was performed using PCR reverse dot blot assay with the thalassemia gene detection kit (Yaneng Biosciences, Shenzhen, China) following the manufacturer's instructions.^6^

### 2.4 Rare genotype test

‐‐^THAI^ genotype was tested using gap polymerase chain reaction (gap‐PCR), and HKαα genotype was tested by single PCR and nested PCR, as described previously.^7^, ^8^ For suspected rare types of α‐thalassemia, the full‐length α1‐globin genes and the full‐length α2‐globin genes were amplified using PCR assay and checked. The purified PCR products were subjected to direct sequencing with an ABI 3100 DNA Sequencer (Applied Biosystems; Foster City, CA, USA) as described.^9^

### 2.5 Statistical analysis

All data were entered into and managed using Microsoft Excel 2007 (Microsoft; Redmond, WA, USA). The data were analyzed using the Statistical Package for the Social Sciences (SPSS) version 25 (IBM Inc., Chicago, USA). Associations between hematological indices and the variant genotypes were assessed by a nonparametric Kruskal–Wallis test, presented as median (95% confidence interval). The results with *p* < 0.05 were considered statistically significant.

## 3 RESULTS

### 3.1 Genotypes of common and rare types of α‐thalassemia

Among 10,350 patients, 2923 patients with α‐thalassemia mutation were detected, with a detection rate of 28.24%, including 2666 cases (91.21%) of α deletional genotype, 181 cases (6.19%) of α mutational genotype, 12 cases (0.41%) of α deletion combined with mutation, and 64 cases (2.19%) of α/β complex mutants.

In 2923 patients with α‐thalassemia mutation, the common genotype accounted for 96.00% of all genotypes, mainly including ‐‐^SEA^/αα (64.80%), ‐α^3.7^/αα (18.95%), ‐α^4.2^/αα (4.96%), α^QS^α/αα (3.63%), α^CS^α/αα (1.47%), ‐‐^SEA^/‐α^3.7^ (1.20%), and α^WS^α/αα (0.99%). In addition, rare α‐thalassemia genotypes including ‐‐^THAI^/αα (0.41%), HKαα/‐‐^SEA^ (0.03%), and the novel α‐thalassemia gene mutation CD5 (GCC>ACC) (HGVS named HBA1: c.16G>A) (0.03%) were detected in Fujian province (Table 1).

**TABLE 1:** Genotyping of α‐thalassemia in Fujian province

	Genotype	Phenotype	No. patients detected	Constituent ratio (%)
Common α‐thalassemia	‐‐^SEA^/αα	α^0^/α	1894	64.80
	‐α^3.7^/αα	α^+^/α	554	18.95
	‐α^4.2^/αα	α^+^/α	145	4.96
	‐‐^SEA^/‐α^3.7^	α^0^/α^+^	35	1.20
	‐‐^SEA^/‐α^4.2^	α^0^/α^+^	12	0.41
	‐α^3.7^/‐α^4.2^	α^+^/α^+^	2	0.07
	‐α^3.7^/‐α^3.7^	α^+^/α^+^	11	0.38
	α^QS^α/αα	α^+^/α	106	3.63
	α^CS^α/αα	α^+^/α	43	1.47
	α^WS^α/αα	α^+^/α	29	0.99
	α^QS^α/α^QS^α	α^+^/α^+^	1	0.03
	α^CS^α/α^CS^α	α^+^/α^+^	1	0.03
	‐α^3.7^/α^QS^α	α^+^/α^+^	2	0.07
	‐α^3.7^/α^WS^α	α^+^/α^+^	2	0.07
	‐α^4.2^/α^QS^α	α^+^/α^+^	1	0.03
	‐‐^SEA^/α^QS^α	α^0^/α^+^	4	0.14
	‐‐^SEA^/α^CS^α	α^0^/α^+^	1	0.03
	‐‐^SEA^/α^WS^α	α^0^/α^+^	2	0.07
Subtotal		—	2845	97.33
Rare α‐thalassemia	‐‐^THAI^/αα	α^0^/α	12	0.41
	HKαα/‐‐^SEA^	α^+^/α^0^	1	0.03
	CD5 (GCC>ACC)	α^+^/α	1	0.03
Subtotal		—	14	0.48
Common concurrent α‐and β‐thalassemia	‐α^3.7^/αα/β^CD41–42(‐TCTT)^/β^N^	α^+^/α/β^0^/β^N^	4	0.14
	‐‐^SEA^/αα/β^IVS‐2‐654(C→T)^/β^N^	α^0^/α/β^+^/β^N^	11	0.38
	α^WS^α/αα/β^IVS‐2‐654(C→T)^/β^N^	α^+^/α/β^+^/β^N^	1	0.03
	‐‐^SEA^/‐α^4.2^/β^CD41–42(‐TCTT)^/β^N^	α^0^/α^+^/β^0^/β^N^	1	0.03
	‐α^3.7^/αα/β^IVS‐2‐654(C→T)^/β^N^	α^+^/α/β^+^/β^N^	11	0.38
	‐α^3.7^/αα/β^CAP + 40‐ + 43(‐AAAC)^/β^N^	α^+^/α/β^+^/β^N^	1	0.03
	‐‐^SEA^/‐α^3.7^/β^CD41–42(‐TCTT)^/β^N^	α^0^/α^+^/β^0^/β^N^	1	0.03
	‐α^4.2^/αα/β^CD17(A→T)^/β^N^	α^+^/α/β^0^/β^N^	1	0.03
	‐‐^SEA^/αα/β^CD17(A→T)^/β^N^	α^0^/α/β^0^/β^N^	3	0.10
	‐‐^SEA^/αα/β^‐28(A→G)^/β^N^	α^0^/α/β^+^/β^N^	4	0.14
	‐‐^SEA^/αα/β^CD41‐42(‐TCTT)^/β^N^	α^0^/α/β^0^/β^N^	11	0.38
	‐‐^SEA^/‐α^3.7^/β^IVS‐2‐654(C→T)^/β^N^	α^0^/α^+^/β^+^/β^N^	1	0.03
	‐‐^SEA^/αα/β^CD27–28(+C)^/β^N^	α^0^/α/β^0^/β^N^	3	0.10
	‐‐^SEA^/αα/β^CAP + 40‐ + 43(‐AAAC)^/β^N^	α^0^/α/β^+^/β^N^	1	0.03
	‐α^3.7^/αα/β^CD17(A→T)^/β^N^	α^+^/α/β^0^/β^N^	2	0.07
	‐α^3.7^/αα/β^CD71–72(+A)^/β^N^	α^+^/α/β^0^/β^N^	2	0.07
	‐α^4.2^/αα/β^CD41–42(‐TCTT)^/β^N^	α^+^/α/β^0^/β^N^	2	0.07
	α^CS^α/αα/β^CD17(A→T)^/β^N^	α^+^/α/β^0^/β^N^	1	0.03
	α^CS^α/αα/β^IVS‐2‐654(C→T)^/β^N^	α^+^/α/β^+^/β^N^	1	0.03
	‐‐^SEA^/αα/β^CD26(GAG→AAG)^/β^N^	α^0^/α/βE/β^N^	1	0.03
	‐α^3.7^/αα/β^−28(A→G)^/β^N^	α^+^/α/β^+^/β^N^	1	0.03
Subtotal		—	64	2.19
Total		—	2923	100.00

*Note*: α^0^ indicates absent synthesis of α‐globin peptide chain; α^+^ indicates reduced synthesis of α‐globin peptide chain; α indicates no mutation. β^0^ indicates absent synthesis of β‐globin peptide chain; β^+^ indicates reduced synthesis of β‐globin peptide chain; N indicates no mutation.

Abbreviations: CS, Hb Constant Spring; HK, Hong Kong deletion; QS, Hb Quong Sze; SEA, Southeast Asian deletion; THAI, Thailand deletion; WS, Hb Westmead.

### 3.2 Hematological indices, the hemoglobin components and levels, and SF of patients with different genotypes of α‐thalassemia

Hematological indices, the hemoglobin components and levels, and SF concentration in patients with different α‐thalassemia genotypes were analyzed in this study, and we found that the red blood cell count (RBC) in patients with deletional genotypes of α‐thalassemia was higher than that in patients with non‐deletional genotypes of α‐thalassemia (5.3 (5.0, 5.8) × 10^12^/ L vs 4.9 (4.5, 5.4) × 10^12^/L) (*p* < 0.05).Conversely, the levels of Hb, MCV, MCH, and HbA2 in patients with deletional genotypes of α‐thalassemia were lower than that in patients with non‐deletional genotypes of α‐thalassemia (112.0 (106.0, 120.0) g/L vs 119.0 (111.0, 126.8) g/L, 67.5 (65.0, 69.5) fl vs 76.1 (72.2, 79.5) fl, 21.2 (20.5, 21.9) pg vs 24.7 (23.0, 26.1) pg, and 2.3 (2.2, 2.5) % vs 2.5 (2.4, 2.7) %) (*p* < 0.05). (Table 2).

**TABLE 2:** Comparison of hematological indices between three common deletional genotypes and three common non‐deletional genotypes of α‐thalassemia patients

Genotype	No.	Gender (F/M)	Age (year)	RBC (×10^12^/L)	Hb (g/L)	MCV (fl)	MCH (pg)	HbA2 (%)	HbF (%)	SF (μg/L)
Deletional genotype	2593	1839/754	27.0 (5.0, 31.0)	5.3 (5.0, 5.8)	112.0 (106.0, 120.0)	67.5 (65.0, 69.5)	21.2 (20.5, 21.9)	2.3 (2.2, 2.5)	0.6 (0.2, 0.9)	47.6 (25.0, 89.5)
‐‐^SEA^/αα	1894	1312/582	27.0 (22.0, 31.0)	5.2 (4.9, 5.7)	112.0 (105.0, 122.3)	68.4 (66.6, 69.9)	21.7 (21.0, 22.3)	2.3 (2.2, 2.4)	0.3 (0.1, 0.5)	55.6 (33.5103.6)
‐α^3.7^/αα	554	414/140	28.0 (25.0, 32.0)	4.4 (4.0, 4.8)	115.5 (107.5126.3)	81.6 (79.8, 83.5)	26.8 (26.1, 27.6)	2.5 (2.4, 2.6)	0.2 (0.1, 0.4)	35.3 (13.1, 68.7)
‐α^4.2^/αα	145	113/32	28.0 (25.0, 32.0)	4.4 (4.2, 4.8)	116.0 (106.0, 125.0)	80.8 (78.4, 81.4)	26.2 (25.5, 27.2)	2.5 (2.3, 2.6)	0.4 (0.1, 0.6)	47.8 (18.6114.2)
Non‐deletional genotype	178	130/48	29.0 (24.0, 32.0)	4.9 (4.5, 5.4)	119.0 (111.0, 126.8)	76.1 (72.2, 79.5)	24.7 (23.0, 26.1)	2.5 (2.4, 2.7)	0.3 (0.1, 0.6)	34.4 (13.8, 59.5)
α^QS^α/αα	106	75/31	28.0 (3.0, 32.0)	4.7 (4.4, 5.0)	114.0 (106.8121.0)	76.0 (75.0, 77.9)	24.3 (23.6, 25.0)	2.6 (2.5, 3.0)	0.2 (0.1, 0.4)	31.8 (16.6, 40.7)
α^CS^α/αα	43	34/9	27.0 (25.5, 31.0)	4.7 (4.3, 5.0)	118.0 (112.0, 127.5)	79.8 (76.8, 81.5)	26.1 (24.6, 27.5)	2.2 (2.1, 2.3)	0.3 (0.1, 0.4)	84.2 (16.5234.7)
α^WS^α/αα	29	21/8	28.5 (25.5, 30.0)	4.5 (4.3, 5.0)	119.0 (104.5126.0)	80.3 (71.6, 81.0)	26.3 (22.3, 27.3)	2.7 (2.5, 3.0)	0.2 (0.1, 0.4)	22.3 (8.8, 57.2)
*A comparison of deletional genotype*										
*p*‐value^a^ *p*‐value^b^ *p*‐value^c^			<0.001*	<0.001*	<0.001*	<0.001*	<0.001*	<0.001*	0.002*	0.002*
			0.002*	<0.001*	<0.001*	<0.001*	<0.001*	<0.001*	0.570	0.071
			0.567	0.523	0.603	0.543	0.686	0.481	0.475	0.732
*A comparison of non‐deletional genotype*										
*p*‐value^d^ *p*‐value^e^ *p*‐value^f^			0.704	0.045*	0.949	<0.001*	<0.001*	<0.001*	0.921	0.288
			0.510	0.250	0.671	0.003*	0.001*	0.670	0.961	0.759
			0.606	0.952	0.444	0.305	0.574	0.001*	0.952	0.859
*A comparison of deletional genotype and non‐deletional genotype*										
*p*‐value^g^			0.509	<0.001*	<0.001*	<0.001*	<0.001*	<0.001*	0.421	0.191

Abbreviations: F, Female; M, Male.

^a^ Subjects with ‐‐^SEA^/αα compared with were subjects with ‐α^3.7^/αα.

^b^ Subjects with ‐‐^SEA^/αα compared with were subjects with ‐α^4.2^/αα.

^c^ Subjects with ‐α^3.7^/αα compared with were subjects with ‐α^4.2^/αα.

^d^ Subjects with α^QS^α/αα compared with were subjects with α^CS^α/αα.

^e^ Subjects with α^QS^α/αα compared with were subjects with α^WS^α/αα.

^f^ Subjects with α^CS^α/αα compared with were subjects with α^WS^α/αα.

^g^ Subjects with deletional genotype compared with were subjects with non‐deletional genotype.

^h^ All hematological indices with more than one records are presented as median (95% confidence interval). Bootstrap method is used in computing 95% confidence intervals.

* *p* < 0.05, Kruskal–Wallis test.

Moreover, some indices in different deletional genotypes of α‐thalassemia and non‐deletional genotypes of α‐thalassemia were various. Among the deletional genotypes of α‐thalassemia, patients with ‐‐^SEA^/αα were found to have higher RBC, HbF, and SF and lower Hb, MCV, MCH, and HbA2 than patients with ‐α^3.7^/αα (*p* < 0.05). What is more, patients with ‐‐^SEA^/αα were found to have higher RBC and lower Hb, MCV, MCH, and HbA2 than patients with ‐α^4.2^/αα (*p* < 0.05). Age of ‐‐^SEA^/αα was lower than that of ‐α^3.7^/αα and ‐α^4.2^/αα (*p* < 0.05) Among the non‐deletional genotypes of α‐thalassemia, patients with α^QS^α/αα were found to have higher RBC and HbA2 and lower MCV and MCH than patients with α^CS^α/αα (*p* < 0.05). Patients with α^QS^α/αα were found to have lower MCV and MCH than patients with α^WS^α/αα (*p* < 0.05). In addition, patients with α^CS^α/αα were found to have lower HbA2 than patients with α^WS^α/αα (*p* < 0.05). (Table 2).

### 3.3 Hematological analysis and molecular diagnosis of the novel α‐thalassemia gene mutation in Fujian province

**FIGURE 1:**
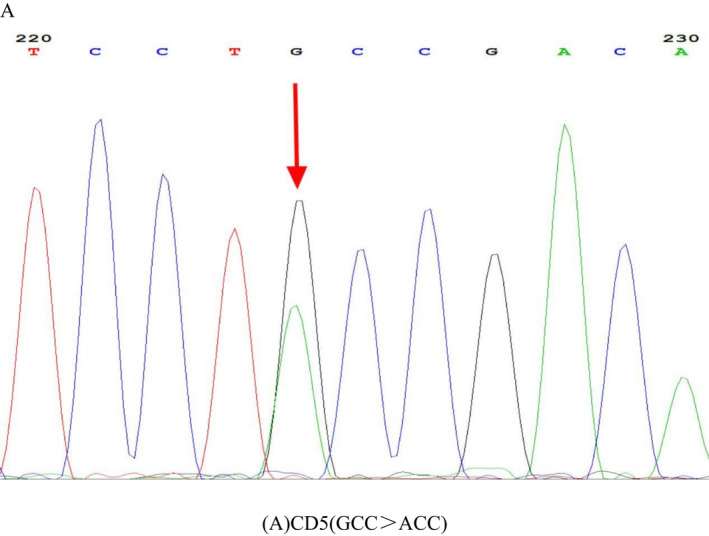
Sequencing of the α1‐globin mutation

In this study, we found a patient showed moderate microcytic hypochromic anemia with Hb 86 g/L, MCV 66.4 fL, and MCH 19.1 pg and decreased level of HbA_2_ (1.9%) that was suspected to be a α‐thalassemia carrier. Sequencing the full‐length α1‐globin gene and of the full‐length α2‐globin gene of the DNA from the peripheral blood sample was done. The results showed there were double peaks at nt224, G>A, in HBA1, corresponding to CD5 (GCC>ACC). It was named HBA1: c.16G>A using the Human Genome Variation Society (HGVS) nomenclature (Table 3, Figure 1).

**TABLE 3:** Hematological indices and genetic results of the patient with α‐thalassemia

Gender	Age (year)	Hb (g/L)	MCV (fl)	MCH (pg)	HbA2 (%)	HbF (%)	HGVS	Mutational genotype
Female	36	86	66.4	19.1	1.9	0	HBA1:c.16G>A	CD5 (GCC>ACC)

## 4 DISCUSSION

α‐thalassemia is common genetic diseases in human and is a specific recessive genetic disease caused by the obstruction of α‐globin peptide chain synthesis.^10^ The correlation between genotype and phenotype in 78 patients with α‐thalassemia in Hainan province was reported,^11^ and our previous study also reported the distribution of genotype in 314 patients with α‐thalassemia in Fujian province.^12^ In this study, we further analyzed the correlation between genotype and phenotype in a large sample of 2923 cases of α‐thalassemia in Fujian province, filling in the gap in this field.

There are two types of α‐thalassemia: deletional genotype and non‐deletional genotype. In addition to the most common deletional genotype, rare deletional genotype of α‐thalassemia has been found in different populations.^13^ Up to now, more than 40 different deletional genotypes of α‐thalassemia have been described, of which 10 types have been found in China.^14^ There are three kinds of deletional genotype of α‐thalassemia in clinic: ‐‐^SEA^/αα, ‐α^3.7^/αα, and ‐α^4.2^/αα. Among 2923 cases with α‐thalassemia, there were 1894 cases (64.80%) with‐‐^SEA^/αα, 554 cases (18.95%) with ‐α^3.7^/αα, and 145 cases (4.96%) with ‐α^4.2^/αα, suggesting that the most common genotype of α ‐thalassemia in this region was ‐‐^SEA^/αα, which was consistent with the reports from Guangxi, Guangdong, Chongqing, and Hainan.^15^ The most common non‐deletional genotypes of α‐thalassemia were α^QS^α/αα (3.63%), followed by α^CS^α/αα (1.47%) and α^WS^α/αα (0.99%), which were the same as those in Chongqing and slightly different from those in Guangxi, Guangdong, and Hainan,^16^, ^17^, ^18^, ^19^ and were speculated to be related to regional population and other genetic factors. The spectrum of mutations detected in this study is similar to that observed in adjacent provinces, but there are differences in genotype prevalence in different regions. The incidence of α‐thalassemia in adjacent provinces of Fujian, such as Guangdong, Guangxi, and Jiangsu, was respectively 8.53%, 15.33%, and 1.48%, and ‐‐^SEA^/αα was dominant in these provinces, and the frequencies were respectively 57.55%, 52.78%, and 83.87%.^20^ This is due to the random distribution of α‐thalassemia in the world, different ethnic regions have their own distribution characteristics. It is generally believed that populations with the same genetic background are prone to homogenous mutations.

Three rare α‐thalassemia genotypes were also detected in this study, including ‐‐^THAI^/αα (12 cases, 0.41%), HKαα/‐‐^SEA^ (1 case, 0.03%), and the novel α‐thalassemia gene mutation CD5 (GCC>ACC) (1 case, 0.03%) in Fujian Province. ‐‐^THAI^ genotype has been gradually found in the population of southern China, mainly distributed in Guangxi, Guangdong, Taiwan, and Fujian.^21^, ^22^, ^23^, ^24^ Like the ‐‐^SEA^ genotype, the phenotype of ‐‐^THAI^ is manifested as microcytic hypochromic anemia and the Thai‐type homozygote or Thai‐type heterozygotes with SEA‐type α‐thalassemia are manifested as Bart's hydrops fetalis.^25^ Because, testing for Thai‐type gene mutation is not routinely performed, which often results in missed diagnosis. Up to now, there are few reports about HKαα/αα type thalassemia, which mainly occurs in Guangxi and Guangdong of China.^21^, ^22^ HKαα allele is a special structure containing ‐α^3.7^ fragment and ααα^anti4.2^ fragment formed by recombination and unequal transposition of the homologous X segment of the α‐globin gene cluster. So far, ααα^anti4.2^ fragments cannot be detected through the routine thalassemia diagnostic kit. The genotype of ‐α^3.7^/αα, HKαα/‐α^3.7^, and HKαα/αα is entirely the result of ‐α^3.7^/αα by gap‐PCR.^26^ The clinical symptoms of HKαα/αα were much milder than those of ‐α^3.7^/αα. Therefore, the symptoms of thalassemia associated with HKαα/αα and ‐‐^SEA^ were milder than those of hemoglobin H disease associated with ‐α^3.7^ and ‐‐^SEA^, and only mild globin production disorder was observed (usually Hb > 100 g/L). If both husband and wife carry HKαα/αα and ‐‐^SEA^ respectively, the fetus can be kept. However, if the HKαα/αα or anti‐HKαα/αα genotypes are incorrectly identified as ‐α^3.7^/αα, it may lead to incorrect clinical treatment.^27^ HBA1: c.16G>A mutation was first reported in Fujian Province, which was discovered in Shenzhen.^28^ In their report, the phenotype of this mutation was normal and only with the decreased level of HbA2, considered as the polymorphism site of HBA1. However, in our study, the patient with this mutation showed moderate anemia symptoms, with Hb 86 g/L, MCV 66.4 fl, MCH 19.1 pg, and HbA2 1.9%, which might be due to the fact that the patient, who was pregnant, might show blood volume change^29^ and the patient was found to be diagnosed as iron deficiency anemia few days later, when follow‐up.

In addition, 64 cases (2.19%) with concurrent α‐ and β‐thalassemia were detected in 2923 positive samples. Patients with concurrent α‐ and β‐thalassemia have been reported to suffer from mild anemia due to a reduction in α‐ and β‐globin chain synthesis, which alleviates the imbalance caused by reduced globin chain synthesis and thus reduces the severity of anemia.^30^ In this study, we detected 2.19% concurrent α‐ and β‐thalassemia, the five most common genotypes including ‐‐^SEA^/αα/β^IVS‐2‐654(C→T)^/β^N^ (11 cases), ‐α^3.7^/αα/β^IVS‐2‐654(C→T)^/β^N^ (11 cases), ‐‐^SEA^/αα/β^CD41‐42(‐CTTT)^/β^N^ (11 cases), ‐α^3.7^/αα/β^CD41‐42(‐CTTT)^/β^N^ (4 cases), and ‐‐^SEA^/αα/β^−28(A→G)^/β^N^ (4 cases). While patients with α‐ and β‐thalassemia have milder symptoms, their offspring are more likely than the general population to develop severe thalassemia, and the long‐term damage is much greater. Therefore, the clinical diagnosis of concurrent α‐ and β‐thalassemia should not be ignored. According to Xiong Fu et al.,^31^ concurrent α‐ and β‐thalassemia is generally manifested as the phenotypic characteristics of β‐thalassemia in Hb analysis, so α‐thalassemia is easy to be missed and misdiagnosed in clinical diagnosis, which should be paid attention to.

There is significant variation in clinical severity among patients with α‐thalassemia, which is indirectly reflected by the span of age at first diagnosis. Age of ‐‐^SEA^/αα was lower than that of ‐α^3.7^/αα and ‐α^4.2^/αα (*p* < 0.05); this may be related to the number of inactivated α‐globin genes of ‐‐^SEA^/αα were more than ‐α^3.7^/αα and ‐α^4.2^/αα. Patients with ‐‐^SEA^/αα were associated with more severe anemia and came to the hospital earlier for diagnosis and treatment. Chen Suqin et al.^32^ also demonstrated that factors such as age influenced the genotype and phenotype of α‐thalassemia. Liebhaber et al.^33^ found that the α1‐globin gene on the same chromosome as the Hb CS mutation was only half as expressed as the ‐α^4.2^ or ‐α^3.7^ variants. Thus, the absence of α‐globin peptide chains or the relative excess of β‐globin peptide chains is more severe in non‐deletional patients. The hematologic appearance of patients with non‐deletional genotype of α‐thalassemia differs from that patients with deletional genotype of α‐thalassemia; anemia is more severe in patients with non‐deletional genotype of α‐thalassemia.^34^ In our study, patients with deletional genotypes of α‐thalassemia were found to have higher RBC and lower Hb, MCV, MCH, and HbA2 than patients with non‐deletional genotypes of α‐thalassemia (*p* < 0.05).It may be related to the damage of erythrocyte membrane caused by excess β‐chain oxidation and α^QS^α or α^CS^α chain oxidation. This results in decreased EPO production and RBC in non‐deletional genotypes of α‐thalassemia.^35^ α‐thalassemia mainly affects the synthesis of Hb, which is the main content of red blood cells. Therefore, Hb, MCV, and MCH will show obvious changes. Interestingly, we found that α^QS^α/αα's Hb was lower than ‐α^3.7^/αα and ‐α^4.2^/αα, which was partly supported that anemia in the non‐deletional genotype was more severe than in the deletional genotype. Patients with ‐‐^SEA^/αα were found to have higher RBC, HbF, and SF, it may be related to ‐‐^SEA^/αα was associated with more γ‐globin mutations, and its RBC synthesis requires more iron, so the RBC, HbF, and SF were higher than ‐α^3.7^/αα.^36^ The Hb, HbA2, MCV, and MCH of ‐‐^SEA^/αα were lower than ‐α^3.7^/αα and ‐α^4.2^/αα in the three common deletional genotypes, suggesting that the severity of anemia depends on the number of inactivated α‐globin genes.

In conclusion, the clinical manifestations and hematologic phenotypes of α ‐thalassemia are related to genotype. The clinical phenotype of α‐thalassemia is influenced by molecular mechanisms. HBA1: c.16G>A is a novel mutation that was first reported in Fujian province. The discovery of this novel mutation in α‐globin gene has enriched the database of hemoglobin variants, and the detailed genetic analysis and clinical symptom description of this mutation will contribute to the further study of hemoglobin function in the future.

## AUTHOR CONTRIBUTIONS

Yali Pan, Meihuan Chen, Na Lin, Liangpu Xu, and Hailong Huang designed and prepared the study. YanHong Zhang, Min Zhang, and Lingji Chen collected the literature and the data and prepared the study. All authors approved the final study.

## FUNDING INFORMATION

This work was funded by the National Natural Science Foundation of China (no.81970170), the National Natural Science Foundation of Fujian Province (no.2019J01510), and the Fujian provincial health technology project (no.2018‐1‐21).

## CONFLICT OF INTEREST

The authors confirm that they have no competing interests.

## Data Availability

The datasets used and/or analyzed during the current study are available from the corresponding author upon reasonable request.
